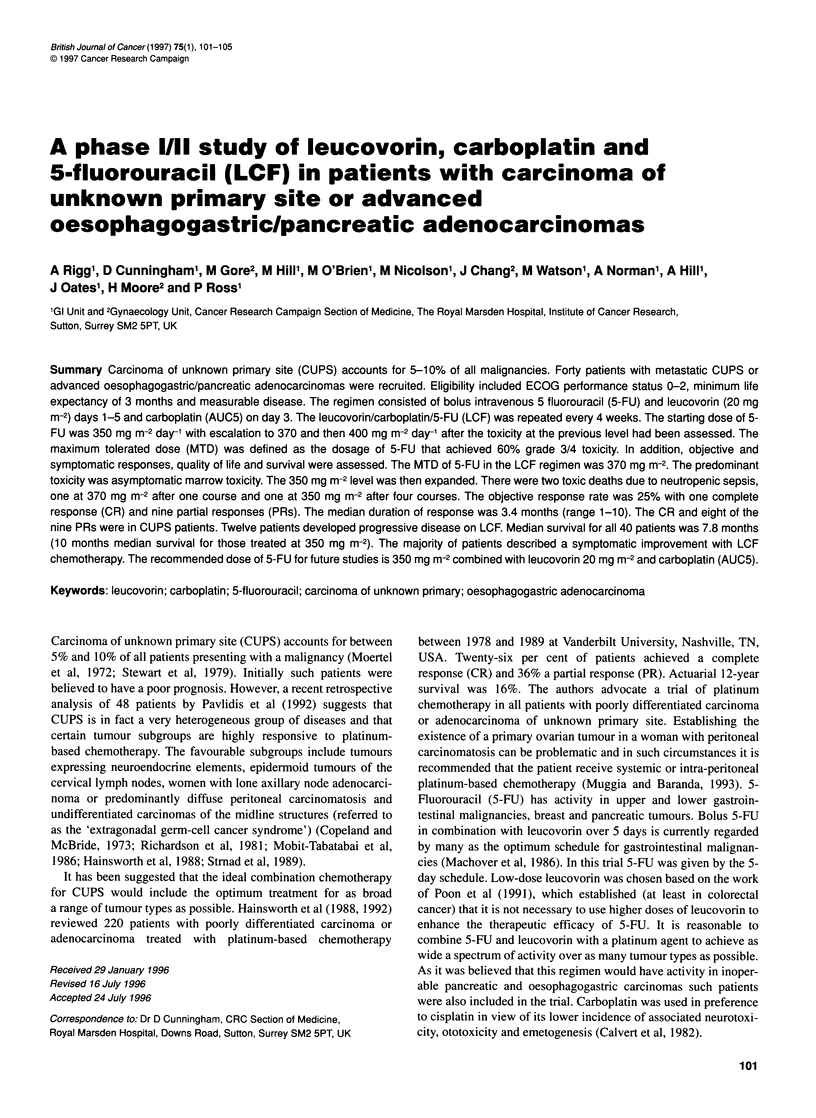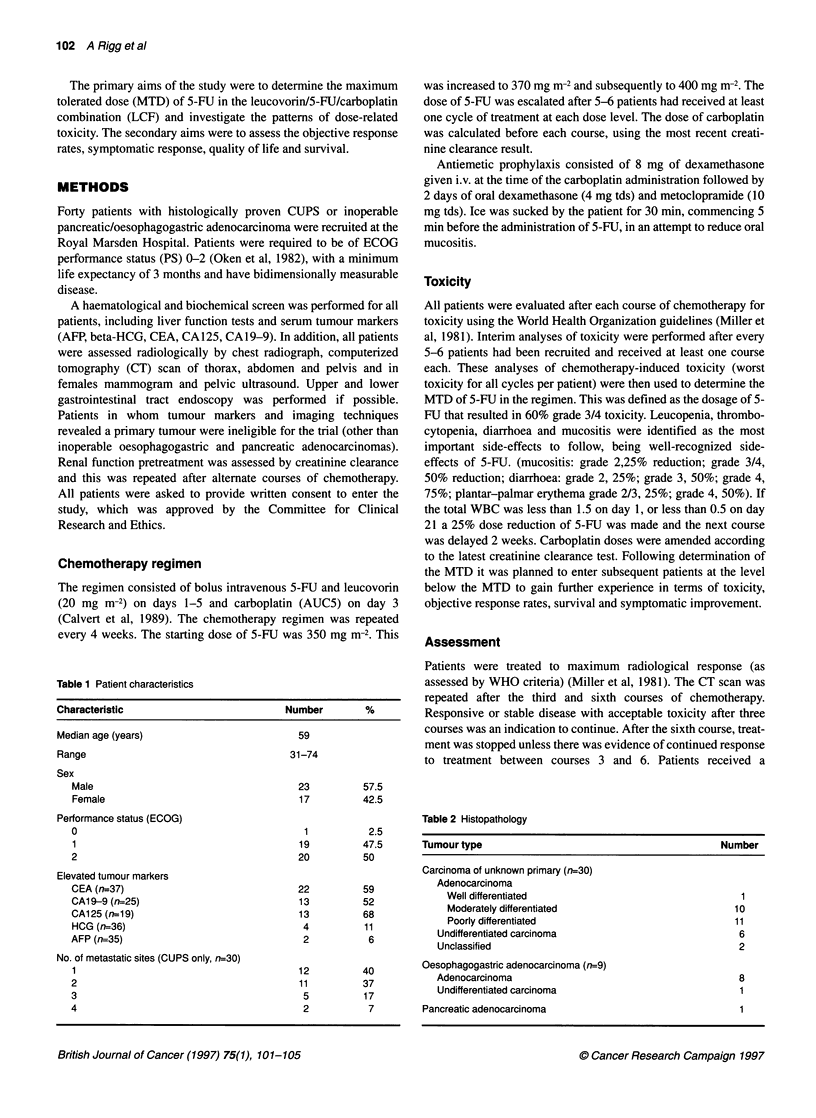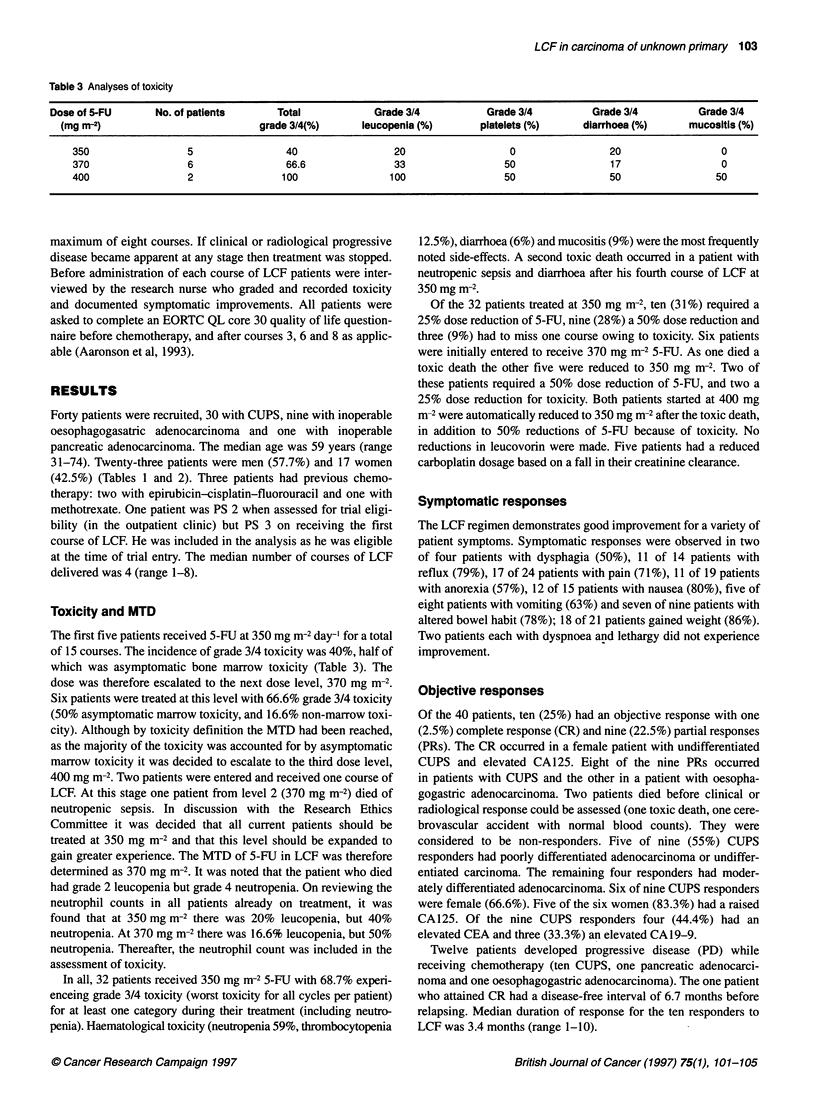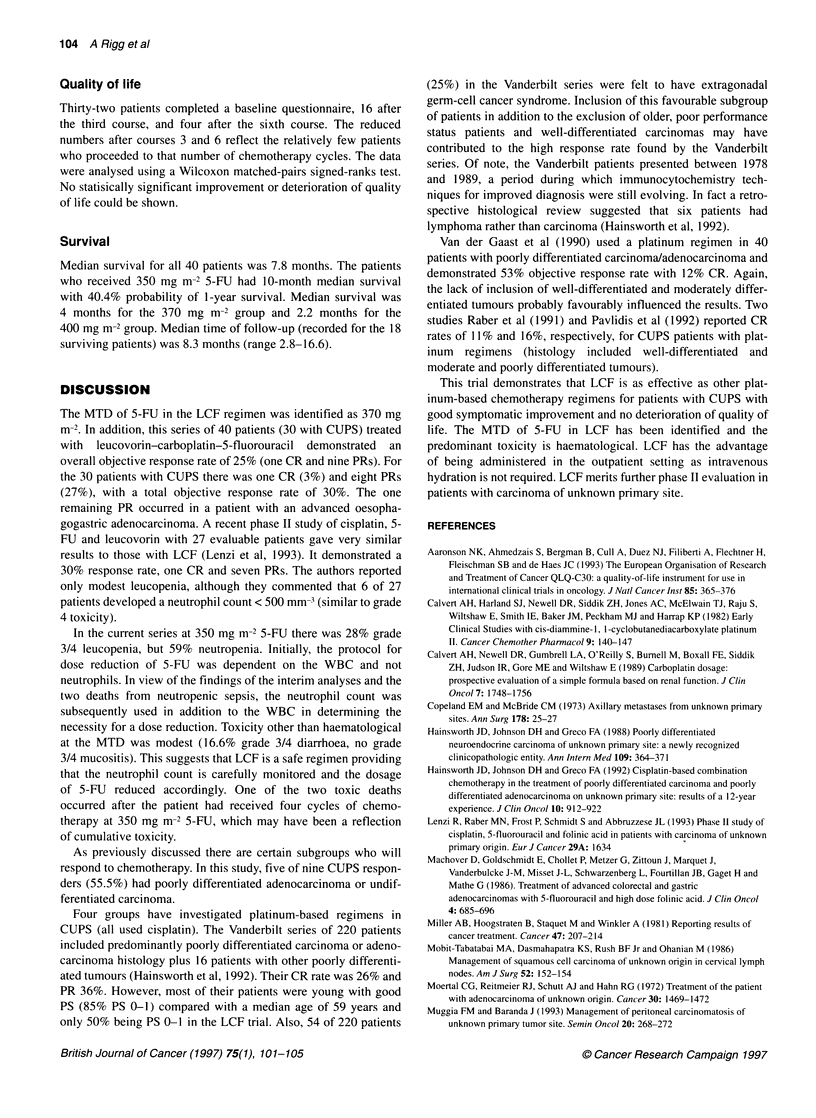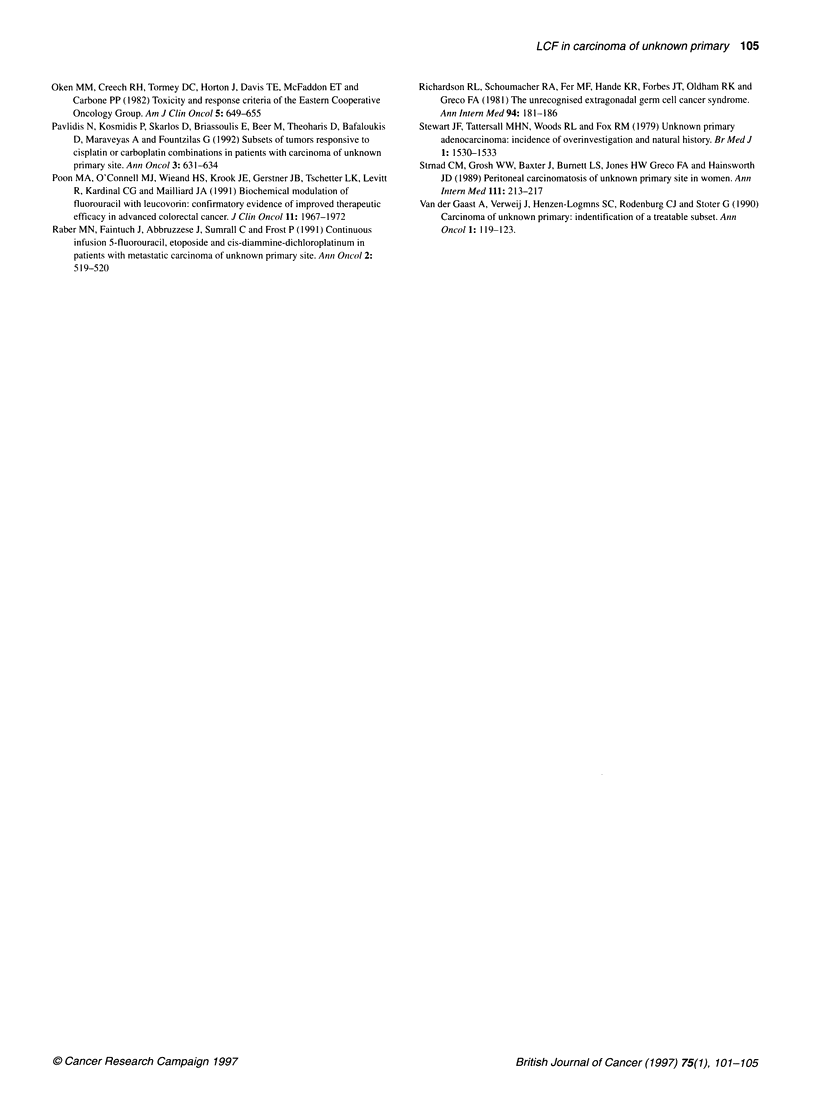# A phase I/II study of leucovorin, carboplatin and 5-fluorouracil (LCF) in patients with carcinoma of unknown primary site or advanced oesophagogastric/pancreatic adenocarcinomas.

**DOI:** 10.1038/bjc.1997.16

**Published:** 1997

**Authors:** A. Rigg, D. Cunningham, M. Gore, M. Hill, M. O'Brien, M. Nicolson, J. Chang, M. Watson, A. Norman, A. Hill, J. Oates, H. Moore, P. Ross

**Affiliations:** GI Unit, Cancer Research Campaign Section of Medicine, The Royal Marsden Hospital, Institute of Cancer Research, Sutton, Surrey, UK.

## Abstract

Carcinoma of unknown primary site (CUPS) accounts for 5-10% of all malignancies. Forty patients with metastatic CUPS or advanced oesophagogastric/pancreatic adenocarcinomas were recruited. Eligibility included ECOG performance status 0-2, minimum life expectancy of 3 months and measurable disease. The regimen consisted of bolus intravenous 5 fluorouracil (5-FU) and leucovorin (20 mg m-2) days 1-5 and carboplatin (AUC5) on day 3. The leucovorin/carboplatin/5-FU (LCF) was repeated every 4 weeks. The starting dose of 5-FU was 350 mg m-2 day-1 with escalation to 370 and then 400 mg m-2 day -1 after the toxicity at the previous level had been assessed. The maximum tolerated dose (MTD) was defined as the dosage of 5-FU that achieved 60% grade 3/4 toxicity. In addition, objective and symptomatic responses, quality of life and survival were assessed. The MTD of 5-FU in the LCF regimen was 370 mg m-2. The predominant toxicity was asymptomatic marrow toxicity. The 350 mg m-2 level was then expanded. There were two toxic deaths due to neutropenic sepsis, one at 370 mg m-2 after one course and one at 350 mg m-2 after four courses. The objective response rate was 25% with one complete response (CR) and nine partial responses (PRs). The median duration of response was 3.4 months (range 1-10). The CR and eight of the nine PRs were in CUPS patients. Twelve patients developed progressive disease on LCF. Median survival for all 40 patients was 7.8 months (10 months median survival for those treated at 350 mg m-2). The majority of patients described a symptomatic improvement with LCF chemotherapy. The recommended dose of 5-FU for future studies is 350 mg m-2 combined with leucovorin 20 mg m-2 and carboplatin (AUC5).


British Journal of Cancer (1997) 75(1), 101–105
© 1997 Cancer Research Campaign

### A phase I/II study of leucovorin, carboplatin and 5-fluorouracil (LCF) in patients with carcinoma of unknown primary site or advanced oesophagogastric/pancreatic adenocarcinomas

A Rigg[1], D Cunningham[1], M Gore[2], M Hill[1], M O'Brien[1], M Nicolson[1], J Chang[2], M Watson[1], A Norman[1], A Hill[1], J Oates[1], H Moore[2] and P Ross[1]

[1]GI Unit and [2]Gynaecology Unit, Cancer Research Campaign Section of Medicine, The Royal Marsden Hospital, Institute of Cancer Research, Sutton, Surrey SM2 5PT, UK

**Summary** Carcinoma of unknown primary site (CUPS) accounts for 5–10% of all malignancies. Forty patients with metastatic CUPS or advanced oesophagogastric/pancreatic adenocarcinomas were recruited. Eligibility included ECOG performance status 0–2, minimum life expectancy of 3 months and measurable disease. The regimen consisted of bolus intravenous 5 fluorouracil (5-FU) and leucovorin (20 mg $m^{-2}$) days 1–5 and carboplatin (AUC5) on day 3. The leucovorin/carboplatin/5-FU (LCF) was repeated every 4 weeks. The starting dose of 5-FU was 350 mg $m^{-2}$ $day^{-1}$ with escalation to 370 and then 400 mg $m^{-2}$ $day^{-1}$ after the toxicity at the previous level had been assessed. The maximum tolerated dose (MTD) was defined as the dosage of 5-FU that achieved 60% grade 3/4 toxicity. In addition, objective and symptomatic responses, quality of life and survival were assessed. The MTD of 5-FU in the LCF regimen was 370 mg $m^{-2}$. The predominant toxicity was asymptomatic marrow toxicity. The 350 mg $m^{-2}$ level was then expanded. There were two toxic deaths due to neutropenic sepsis, one at 370 mg $m^{-2}$ after one course and one at 350 mg $m^{-2}$ after four courses. The objective response rate was 25% with one complete response (CR) and nine partial responses (PRs). The median duration of response was 3.4 months (range 1–10). The CR and eight of the nine PRs were in CUPS patients. Twelve patients developed progressive disease on LCF. Median survival for all 40 patients was 7.8 months (10 months median survival for those treated at 350 mg $m^{-2}$). The majority of patients described a symptomatic improvement with LCF chemotherapy. The recommended dose of 5-FU for future studies is 350 mg $m^{-2}$ combined with leucovorin 20 mg $m^{-2}$ and carboplatin (AUC5).

**Keywords**: leucovorin; carboplatin; 5-fluorouracil; carcinoma of unknown primary; oesophagogastric adenocarcinoma

Carcinoma of unknown primary site (CUPS) accounts for between 5% and 10% of all patients presenting with a malignancy (Moertel et al, 1972; Stewart et al, 1979). Initially such patients were believed to have a poor prognosis. However, a recent retrospective analysis of 48 patients by Pavlidis et al (1992) suggests that CUPS is in fact a very heterogeneous group of diseases and that certain tumour subgroups are highly responsive to platinum-based chemotherapy. The favourable subgroups include tumours expressing neuroendocrine elements, epidermoid tumours of the cervical lymph nodes, women with lone axillary node adenocarcinoma or predominantly diffuse peritoneal carcinomatosis and undifferentiated carcinomas of the midline structures (referred to as the 'extragonadal germ-cell cancer syndrome') (Copeland and McBride, 1973; Richardson et al, 1981; Mobit-Tabatabai et al, 1986; Hainsworth et al, 1988; Strnad et al, 1989).

It has been suggested that the ideal combination chemotherapy for CUPS would include the optimum treatment for as broad a range of tumour types as possible. Hainsworth et al (1988, 1992) reviewed 220 patients with poorly differentiated carcinoma or adenocarcinoma treated with platinum-based chemotherapy between 1978 and 1989 at Vanderbilt University, Nashville, TN, USA. Twenty-six per cent of patients achieved a complete response (CR) and 36% a partial response (PR). Actuarial 12-year survival was 16%. The authors advocate a trial of platinum chemotherapy in all patients with poorly differentiated carcinoma or adenocarcinoma of unknown primary site. Establishing the existence of a primary ovarian tumour in a woman with peritoneal carcinomatosis can be problematic and in such circumstances it is recommended that the patient receive systemic or intra-peritoneal platinum-based chemotherapy (Muggia and Baranda, 1993). 5-Fluorouracil (5-FU) has activity in upper and lower gastrointestinal malignancies, breast and pancreatic tumours. Bolus 5-FU in combination with leucovorin over 5 days is currently regarded by many as the optimum schedule for gastrointestinal malignancies (Machover et al, 1986). In this trial 5-FU was given by the 5-day schedule. Low-dose leucovorin was chosen based on the work of Poon et al (1991), which established (at least in colorectal cancer) that it is not necessary to use higher doses of leucovorin to enhance the therapeutic efficacy of 5-FU. It is reasonable to combine 5-FU and leucovorin with a platinum agent to achieve as wide a spectrum of activity over as many tumour types as possible. As it was believed that this regimen would have activity in inoperable pancreatic and oesophagogastric carcinomas such patients were also included in the trial. Carboplatin was used in preference to cisplatin in view of its lower incidence of associated neurotoxicity, ototoxicity and emetogenesis (Calvert et al, 1982).

*Received 29 January 1996*
*Revised 16 July 1996*
*Accepted 24 July 1996*

*Correspondence to:* Dr D Cunningham, CRC Section of Medicine, Royal Marsden Hospital, Downs Road, Sutton, Surrey SM2 5PT, UK

The primary aims of the study were to determine the maximum tolerated dose (MTD) of 5-FU in the leucovorin/5-FU/carboplatin combination (LCF) and investigate the patterns of dose-related toxicity. The secondary aims were to assess the objective response rates, symptomatic response, quality of life and survival.

### METHODS

Forty patients with histologically proven CUPS or inoperable pancreatic/oesophagogastric adenocarcinoma were recruited at the Royal Marsden Hospital. Patients were required to be of ECOG performance status (PS) 0–2 (Oken et al, 1982), with a minimum life expectancy of 3 months and have bidimensionally measurable disease.

A haematological and biochemical screen was performed for all patients, including liver function tests and serum tumour markers (AFP, beta-HCG, CEA, CA125, CA19–9). In addition, all patients were assessed radiologically by chest radiograph, computerized tomography (CT) scan of thorax, abdomen and pelvis and in females mammogram and pelvic ultrasound. Upper and lower gastrointestinal tract endoscopy was performed if possible. Patients in whom tumour markers and imaging techniques revealed a primary tumour were ineligible for the trial (other than inoperable oesophagogastric and pancreatic adenocarcinomas). Renal function pretreatment was assessed by creatinine clearance and this was repeated after alternate courses of chemotherapy. All patients were asked to provide written consent to enter the study, which was approved by the Committee for Clinical Research and Ethics.

### Chemotherapy regimen

The regimen consisted of bolus intravenous 5-FU and leucovorin (20 mg $m^{-2}$) on days 1–5 and carboplatin (AUC5) on day 3 (Calvert et al, 1989). The chemotherapy regimen was repeated every 4 weeks. The starting dose of 5-FU was 350 mg $m^{-2}$. This was increased to 370 mg $m^{-2}$ and subsequently to 400 mg $m^{-2}$. The dose of 5-FU was escalated after 5–6 patients had received at least one cycle of treatment at each dose level. The dose of carboplatin was calculated before each course, using the most recent creatinine clearance result.

Antiemetic prophylaxis consisted of 8 mg of dexamethasone given i.v. at the time of the carboplatin administration followed by 2 days of oral dexamethasone (4 mg tds) and metoclopramide (10 mg tds). Ice was sucked by the patient for 30 min, commencing 5 min before the administration of 5-FU, in an attempt to reduce oral mucositis.

### Toxicity

All patients were evaluated after each course of chemotherapy for toxicity using the World Health Organization guidelines (Miller et al, 1981). Interim analyses of toxicity were performed after every 5–6 patients had been recruited and received at least one course each. These analyses of chemotherapy-induced toxicity (worst toxicity for all cycles per patient) were then used to determine the MTD of 5-FU in the regimen. This was defined as the dosage of 5-FU that resulted in 60% grade 3/4 toxicity. Leucopenia, thrombocytopenia, diarrhoea and mucositis were identified as the most important side-effects to follow, being well-recognized side-effects of 5-FU. (mucositis: grade 2,25% reduction; grade 3/4, 50% reduction; diarrhoea: grade 2, 25%; grade 3, 50%; grade 4, 75%; plantar–palmar erythema grade 2/3, 25%; grade 4, 50%). If the total WBC was less than 1.5 on day 1, or less than 0.5 on day 21 a 25% dose reduction of 5-FU was made and the next course was delayed 2 weeks. Carboplatin doses were amended according to the latest creatinine clearance test. Following determination of the MTD it was planned to enter subsequent patients at the level below the MTD to gain further experience in terms of toxicity, objective response rates, survival and symptomatic improvement.

### Assessment

Patients were treated to maximum radiological response (as assessed by WHO criteria) (Miller et al, 1981). The CT scan was repeated after the third and sixth courses of chemotherapy. Responsive or stable disease with acceptable toxicity after three courses was an indication to continue. After the sixth course, treatment was stopped unless there was evidence of continued response to treatment between courses 3 and 6. Patients received a

**Table 1** Patient characteristics

| Characteristic | Number | % |
|---|---|---|
| Median age (years) | 59 | |
| Range | 31–74 | |
| Sex | | |
| Male | 23 | 57.5 |
| Female | 17 | 42.5 |
| Performance status (ECOG) | | |
| 0 | 1 | 2.5 |
| 1 | 19 | 47.5 |
| 2 | 20 | 50 |
| Elevated tumour markers | | |
| CEA (*n*=37) | 22 | 59 |
| CA19–9 (*n*=25) | 13 | 52 |
| CA125 (*n*=19) | 13 | 68 |
| HCG (*n*=36) | 4 | 11 |
| AFP (*n*=35) | 2 | 6 |
| No. of metastatic sites (CUPS only, *n*=30) | | |
| 1 | 12 | 40 |
| 2 | 11 | 37 |
| 3 | 5 | 17 |
| 4 | 2 | 7 |

**Table 2** Histopathology

| Tumour type | Number |
|---|---|
| Carcinoma of unknown primary (*n*=30) | |
| Adenocarcinoma | |
| Well differentiated | 1 |
| Moderately differentiated | 10 |
| Poorly differentiated | 11 |
| Undifferentiated carcinoma | 6 |
| Unclassified | 2 |
| Oesophagogastric adenocarcinoma (*n*=9) | |
| Adenocarcinoma | 8 |
| Undifferentiated carcinoma | 1 |
| Pancreatic adenocarcinoma | 1 |

© Cancer Research Campaign 1997

**Table 3** Analyses of toxicity

| Dose of 5-FU (mg m$^{-2}$) | No. of patients | Total grade 3/4(%) | Grade 3/4 leucopenia (%) | Grade 3/4 platelets (%) | Grade 3/4 diarrhoea (%) | Grade 3/4 mucositis (%) |
|---|---|---|---|---|---|---|
| 350 | 5 | 40 | 20 | 0 | 20 | 0 |
| 370 | 6 | 66.6 | 33 | 50 | 17 | 0 |
| 400 | 2 | 100 | 100 | 50 | 50 | 50 |

maximum of eight courses. If clinical or radiological progressive disease became apparent at any stage then treatment was stopped. Before administration of each course of LCF patients were interviewed by the research nurse who graded and recorded toxicity and documented symptomatic improvements. All patients were asked to complete an EORTC QL core 30 quality of life questionnaire before chemotherapy, and after courses 3, 6 and 8 as applicable (Aaronson et al, 1993).

### RESULTS

Forty patients were recruited, 30 with CUPS, nine with inoperable oesophagogasatric adenocarcinoma and one with inoperable pancreatic adenocarcinoma. The median age was 59 years (range 31–74). Twenty-three patients were men (57.7%) and 17 women (42.5%) (Tables 1 and 2). Three patients had previous chemotherapy: two with epirubicin–cisplatin–fluorouracil and one with methotrexate. One patient was PS 2 when assessed for trial eligibility (in the outpatient clinic) but PS 3 on receiving the first course of LCF. He was included in the analysis as he was eligible at the time of trial entry. The median number of courses of LCF delivered was 4 (range 1–8).

### Toxicity and MTD

The first five patients received 5-FU at 350 mg m$^{-2}$ day$^{-1}$ for a total of 15 courses. The incidence of grade 3/4 toxicity was 40%, half of which was asymptomatic bone marrow toxicity (Table 3). The dose was therefore escalated to the next dose level, 370 mg m$^{-2}$. Six patients were treated at this level with 66.6% grade 3/4 toxicity (50% asymptomatic marrow toxicity, and 16.6% non-marrow toxicity). Although by toxicity definition the MTD had been reached, as the majority of the toxicity was accounted for by asymptomatic marrow toxicity it was decided to escalate to the third dose level, 400 mg m$^{-2}$. Two patients were entered and received one course of LCF. At this stage one patient from level 2 (370 mg m$^{-2}$) died of neutropenic sepsis. In discussion with the Research Ethics Committee it was decided that all current patients should be treated at 350 mg m$^{-2}$ and that this level should be expanded to gain greater experience. The MTD of 5-FU in LCF was therefore determined as 370 mg m$^{-2}$. It was noted that the patient who died had grade 2 leucopenia but grade 4 neutropenia. On reviewing the neutrophil counts in all patients already on treatment, it was found that at 350 mg m$^{-2}$ there was 20% leucopenia, but 40% neutropenia. At 370 mg m$^{-2}$ there was 16.6% leucopenia, but 50% neutropenia. Thereafter, the neutrophil count was included in the assessment of toxicity.

In all, 32 patients received 350 mg m$^{-2}$ 5-FU with 68.7% experienceing grade 3/4 toxicity (worst toxicity for all cycles per patient) for at least one category during their treatment (including neutropenia). Haematological toxicity (neutropenia 59%, thrombocytopenia 12.5%), diarrhoea (6%) and mucositis (9%) were the most frequently noted side-effects. A second toxic death occurred in a patient with neutropenic sepsis and diarrhoea after his fourth course of LCF at 350 mg m$^{-2}$.

Of the 32 patients treated at 350 mg m$^{-2}$, ten (31%) required a 25% dose reduction of 5-FU, nine (28%) a 50% dose reduction and three (9%) had to miss one course owing to toxicity. Six patients were initially entered to receive 370 mg m$^{-2}$ 5-FU. As one died a toxic death the other five were reduced to 350 mg m$^{-2}$. Two of these patients required a 50% dose reduction of 5-FU, and two a 25% dose reduction for toxicity. Both patients started at 400 mg m$^{-2}$ were automatically reduced to 350 mg m$^{-2}$ after the toxic death, in addition to 50% reductions of 5-FU because of toxicity. No reductions in leucovorin were made. Five patients had a reduced carboplatin dosage based on a fall in their creatinine clearance.

### Symptomatic responses

The LCF regimen demonstrates good improvement for a variety of patient symptoms. Symptomatic responses were observed in two of four patients with dysphagia (50%), 11 of 14 patients with reflux (79%), 17 of 24 patients with pain (71%), 11 of 19 patients with anorexia (57%), 12 of 15 patients with nausea (80%), five of eight patients with vomiting (63%) and seven of nine patients with altered bowel habit (78%); 18 of 21 patients gained weight (86%). Two patients each with dyspnoea and lethargy did not experience improvement.

### Objective responses

Of the 40 patients, ten (25%) had an objective response with one (2.5%) complete response (CR) and nine (22.5%) partial responses (PRs). The CR occurred in a female patient with undifferentiated CUPS and elevated CA125. Eight of the nine PRs occurred in patients with CUPS and the other in a patient with oesophagogastric adenocarcinoma. Two patients died before clinical or radiological response could be assessed (one toxic death, one cerebrovascular accident with normal blood counts). They were considered to be non-responders. Five of nine (55%) CUPS responders had poorly differentiated adenocarcinoma or undifferentiated carcinoma. The remaining four responders had moderately differentiated adenocarcinoma. Six of nine CUPS responders were female (66.6%). Five of the six women (83.3%) had a raised CA125. Of the nine CUPS responders four (44.4%) had an elevated CEA and three (33.3%) an elevated CA19–9.

Twelve patients developed progressive disease (PD) while receiving chemotherapy (ten CUPS, one pancreatic adenocarcinoma and one oesophagogastric adenocarcinoma). The one patient who attained CR had a disease-free interval of 6.7 months before relapsing. Median duration of response for the ten responders to LCF was 3.4 months (range 1–10).

© Cancer Research Campaign 1997

### Quality of life

Thirty-two patients completed a baseline questionnaire, 16 after the third course, and four after the sixth course. The reduced numbers after courses 3 and 6 reflect the relatively few patients who proceeded to that number of chemotherapy cycles. The data were analysed using a Wilcoxon matched-pairs signed-ranks test. No statisically significant improvement or deterioration of quality of life could be shown.

### Survival

Median survival for all 40 patients was 7.8 months. The patients who received 350 mg $m^{-2}$ 5-FU had 10-month median survival with 40.4% probability of 1-year survival. Median survival was 4 months for the 370 mg $m^{-2}$ group and 2.2 months for the 400 mg $m^{-2}$ group. Median time of follow-up (recorded for the 18 surviving patients) was 8.3 months (range 2.8–16.6).

### DISCUSSION

The MTD of 5-FU in the LCF regimen was identified as 370 mg $m^{-2}$. In addition, this series of 40 patients (30 with CUPS) treated with leucovorin–carboplatin–5-fluorouracil demonstrated an overall objective response rate of 25% (one CR and nine PRs). For the 30 patients with CUPS there was one CR (3%) and eight PRs (27%), with a total objective response rate of 30%. The one remaining PR occurred in a patient with an advanced oesophagogastric adenocarcinoma. A recent phase II study of cisplatin, 5-FU and leucovorin with 27 evaluable patients gave very similar results to those with LCF (Lenzi et al, 1993). It demonstrated a 30% response rate, one CR and seven PRs. The authors reported only modest leucopenia, although they commented that 6 of 27 patients developed a neutrophil count < 500 $mm^{-3}$ (similar to grade 4 toxicity).

In the current series at 350 mg $m^{-2}$ 5-FU there was 28% grade 3/4 leucopenia, but 59% neutropenia. Initially, the protocol for dose reduction of 5-FU was dependent on the WBC and not neutrophils. In view of the findings of the interim analyses and the two deaths from neutropenic sepsis, the neutrophil count was subsequently used in addition to the WBC in determining the necessity for a dose reduction. Toxicity other than haematological at the MTD was modest (16.6% grade 3/4 diarrhoea, no grade 3/4 mucositis). This suggests that LCF is a safe regimen providing that the neutrophil count is carefully monitored and the dosage of 5-FU reduced accordingly. One of the two toxic deaths occurred after the patient had received four cycles of chemotherapy at 350 mg $m^{-2}$ 5-FU, which may have been a reflection of cumulative toxicity.

As previously discussed there are certain subgroups who will respond to chemotherapy. In this study, five of nine CUPS responders (55.5%) had poorly differentiated adenocarcinoma or undifferentiated carcinoma.

Four groups have investigated platinum-based regimens in CUPS (all used cisplatin). The Vanderbilt series of 220 patients included predominantly poorly differentiated carcinoma or adenocarcinoma histology plus 16 patients with other poorly differentiated tumours (Hainsworth et al, 1992). Their CR rate was 26% and PR 36%. However, most of their patients were young with good PS (85% PS 0–1) compared with a median age of 59 years and only 50% being PS 0–1 in the LCF trial. Also, 54 of 220 patients (25%) in the Vanderbilt series were felt to have extragonadal germ-cell cancer syndrome. Inclusion of this favourable subgroup of patients in addition to the exclusion of older, poor performance status patients and well-differentiated carcinomas may have contributed to the high response rate found by the Vanderbilt series. Of note, the Vanderbilt patients presented between 1978 and 1989, a period during which immunocytochemistry techniques for improved diagnosis were still evolving. In fact a retrospective histological review suggested that six patients had lymphoma rather than carcinoma (Hainsworth et al, 1992).

Van der Gaast et al (1990) used a platinum regimen in 40 patients with poorly differentiated carcinoma/adenocarcinoma and demonstrated 53% objective response rate with 12% CR. Again, the lack of inclusion of well-differentiated and moderately differentiated tumours probably favourably influenced the results. Two studies Raber et al (1991) and Pavlidis et al (1992) reported CR rates of 11% and 16%, respectively, for CUPS patients with platinum regimens (histology included well-differentiated and moderate and poorly differentiated tumours).

This trial demonstrates that LCF is as effective as other platinum-based chemotherapy regimens for patients with CUPS with good symptomatic improvement and no deterioration of quality of life. The MTD of 5-FU in LCF has been identified and the predominant toxicity is haematological. LCF has the advantage of being administered in the outpatient setting as intravenous hydration is not required. LCF merits further phase II evaluation in patients with carcinoma of unknown primary site.

### REFERENCES

Aaronson NK, Ahmedzais S, Bergman B, Cull A, Duez NJ, Filiberti A, Flechtner H, Fleischman SB and de Haes JC (1993) The European Organisation of Research and Treatment of Cancer QLQ-C30: a quality-of-life instrument for use in international clinical trials in oncology. *J Natl Cancer Inst* **85:** 365–376

Calvert AH, Harland SJ, Newell DR, Siddik ZH, Jones AC, McElwain TJ, Raju S, Wiltshaw E, Smith IE, Baker JM, Peckham MJ and Harrap KP (1982) Early Clinical Studies with cis-diammine-1, 1-cyclobutanediacarboxylate platinum II. *Cancer Chemother Pharmacol* **9:** 140–147

Calvert AH, Newell DR, Gumbrell LA, O'Reilly S, Burnell M, Boxall FE, Siddik ZH, Judson IR, Gore ME and Wiltshaw E (1989) Carboplatin dosage: prospective evaluation of a simple formula based on renal function. *J Clin Oncol* **7:** 1748–1756

Copeland EM and McBride CM (1973) Axillary metastases from unknown primary sites. *Ann Surg* **178:** 25–27

Hainsworth JD, Johnson DH and Greco FA (1988) Poorly differentiated neuroendocrine carcinoma of unknown primary site: a newly recognized clinicopathologic entity. *Ann Intern Med* **109:** 364–371

Hainsworth JD, Johnson DH and Greco FA (1992) Cisplatin-based combination chemotherapy in the treatment of poorly differentiated carcinoma and poorly differentiated adenocarcinoma on unknown primary site: results of a 12-year experience. *J Clin Oncol* **10:** 912–922

Lenzi R, Raber MN, Frost P, Schmidt S and Abbruzzese JL (1993) Phase II study of cisplatin, 5-fluorouracil and folinic acid in patients with carcinoma of unknown primary origin. *Eur J Cancer* **29A:** 1634

Machover D, Goldschmidt E, Chollet P, Metzer G, Zittoun J, Marquet J, Vanderbulcke J-M, Misset J-L, Schwarzenberg L, Fourtillan JB, Gaget H and Mathe G (1986). Treatment of advanced colorectal and gastric adenocarcinomas with 5-fluorouracil and high dose folinic acid. *J Clin Oncol* **4:** 685–696

Miller AB, Hoogstraten B, Staquet M and Winkler A (1981) Reporting results of cancer treatment. *Cancer* **47:** 207–214

Mobit-Tabatabai MA, Dasmahapatra KS, Rush BF Jr and Ohanian M (1986) Management of squamous cell carcinoma of unknown origin in cervical lymph nodes. *Am J Surg* **52:** 152–154

Moertal CG, Reitmeier RJ, Schutt AJ and Hahn RG (1972) Treatment of the patient with adenocarcinoma of unknown origin. *Cancer* **30:** 1469–1472

Muggia FM and Baranda J (1993) Management of peritoneal carcinomatosis of unknown primary tumor site. *Semin Oncol* **20:** 268–272

 © Cancer Research Campaign 1997

Oken MM, Creech RH, Tormey DC, Horton J, Davis TE, McFaddon ET and Carbone PP (1982) Toxicity and response criteria of the Eastern Cooperative Oncology Group. *Am J Clin Oncol* **5:** 649–655

Pavlidis N, Kosmidis P, Skarlos D, Briassoulis E, Beer M, Theoharis D, Bafaloukis D, Maraveyas A and Fountzilas G (1992) Subsets of tumors responsive to cisplatin or carboplatin combinations in patients with carcinoma of unknown primary site. *Ann Oncol* **3:** 631–634

Poon MA, O'Connell MJ, Wieand HS, Krook JE, Gerstner JB, Tschetter LK, Levitt R, Kardinal CG and Mailliard JA (1991) Biochemical modulation of fluorouracil with leucovorin: confirmatory evidence of improved therapeutic efficacy in advanced colorectal cancer. *J Clin Oncol* **11:** 1967–1972

Raber MN, Faintuch J, Abbruzzese J, Sumrall C and Frost P (1991) Continuous infusion 5-fluorouracil, etoposide and cis-diammine-dichloroplatinum in patients with metastatic carcinoma of unknown primary site. *Ann Oncol* **2:** 519–520

Richardson RL, Schoumacher RA, Fer MF, Hande KR, Forbes JT, Oldham RK and Greco FA (1981) The unrecognised extragonadal germ cell cancer syndrome. *Ann Intern Med* **94:** 181–186

Stewart JF, Tattersall MHN, Woods RL and Fox RM (1979) Unknown primary adenocarcinoma: incidence of overinvestigation and natural history. *Br Med J* **1:** 1530–1533

Strnad CM, Grosh WW, Baxter J, Burnett LS, Jones HW Greco FA and Hainsworth JD (1989) Peritoneal carcinomatosis of unknown primary site in women. *Ann Intern Med* **111:** 213–217

Van der Gaast A, Verweij J, Henzen-Logmns SC, Rodenburg CJ and Stoter G (1990) Carcinoma of unknown primary: indentification of a treatable subset. *Ann Oncol* **1:** 119–123.

© Cancer Research Campaign 1997